# Radiomics diagnostic performance for predicting lymph node metastasis in esophageal cancer: a systematic review and meta-analysis

**DOI:** 10.1186/s12880-024-01278-5

**Published:** 2024-06-12

**Authors:** Dong Ma, Teli Zhou, Jing Chen, Jun Chen

**Affiliations:** 1 The Fifth Affiliated Hospital, Southern Medical University, Guangzhou, Guangdong 510900 China; 2 Guangzhou Shiyuan Clinics Co., Ltd, Guangzhou, Guangdong 510530 China; 3 Dingxi People’s Hospital, Dingxi, Gansu 743000 China

**Keywords:** Radiomics, CT-scan, Artificial intelligence, Esophageal cancer, Machine learning, Deep learnin

## Abstract

**Background:**

Esophageal cancer, a global health concern, impacts predominantly men, particularly in Eastern Asia. Lymph node metastasis (LNM) significantly influences prognosis, and current imaging methods exhibit limitations in accurate detection. The integration of radiomics, an artificial intelligence (AI) driven approach in medical imaging, offers a transformative potential. This meta-analysis evaluates existing evidence on the accuracy of radiomics models for predicting LNM in esophageal cancer.

**Methods:**

We conducted a systematic review following PRISMA 2020 guidelines, searching Embase, PubMed, and Web of Science for English-language studies up to November 16, 2023. Inclusion criteria focused on preoperatively diagnosed esophageal cancer patients with radiomics predicting LNM before treatment. Exclusion criteria were applied, including non-English studies and those lacking sufficient data or separate validation cohorts. Data extraction encompassed study characteristics and radiomics technical details. Quality assessment employed modified Quality Assessment of Diagnostic Accuracy Studies (QUADAS-2) and Radiomics Quality Score (RQS) tools. Statistical analysis involved random-effects models for pooled sensitivity, specificity, diagnostic odds ratio (DOR), and area under the curve (AUC). Heterogeneity and publication bias were assessed using Deek’s test and funnel plots. Analysis was performed using Stata version 17.0 and meta-DiSc.

**Results:**

Out of 426 initially identified citations, nine studies met inclusion criteria, encompassing 719 patients. These retrospective studies utilized CT, PET, and MRI imaging modalities, predominantly conducted in China. Two studies employed deep learning-based radiomics. Quality assessment revealed acceptable QUADAS-2 scores. RQS scores ranged from 9 to 14, averaging 12.78. The diagnostic meta-analysis yielded a pooled sensitivity, specificity, and AUC of 0.72, 0.76, and 0.74, respectively, representing fair diagnostic performance. Meta-regression identified the use of combined models as a significant contributor to heterogeneity (p-value = 0.05). Other factors, such as sample size (> 75) and least absolute shrinkage and selection operator (LASSO) usage for feature extraction, showed potential influence but lacked statistical significance (0.05 < p-value < 0.10). Publication bias was not statistically significant.

**Conclusion:**

Radiomics shows potential for predicting LNM in esophageal cancer, with a moderate diagnostic performance. Standardized approaches, ongoing research, and prospective validation studies are crucial for realizing its clinical applicability.

**Supplementary Information:**

The online version contains supplementary material available at 10.1186/s12880-024-01278-5.

## Introduction

Esophageal cancer, ranking seventh globally in incidence and sixth in mortality, affects predominantly men, especially in Eastern Asia, with nearly half a million new cases and deaths. The two main subtypes, squamous cell carcinoma (LUSC) and adenocarcinoma (LUAD), are associated with distinct patterns; SCC may be declining in Asia due to economic progress, while AC is on the rise in high-income countries, linked to factors like excess body weight [1]. Lymph node metastasis (LNM) is a pivotal factor in esophageal cancer prognosis, influencing long-term survival. The complex lymphatic network around the esophagus leads to metastases in the abdomen, mediastinum, and neck. The diverse patterns of LNM in both squamous cell carcinoma and adenocarcinoma, regardless of the primary tumor location, suggest a need for a more tailored and perhaps more aggressive approach in both surgical and radiotherapeutic management of esophageal cancer. The phenomenon of skip metastasis and the presence of metastases in non-adjacent lymph node stations underscore the complexity of lymphatic drainage from the esophagus and the limitations of current staging and treatment paradigms [2]. The gold standard for detecting LNM in esophageal cancer is histopathological examination of surgically resected lymph nodes, providing a definitive diagnosis through microscopic analysis of tissue [3]. Although invasive, this method is unmatched in accuracy. For non-invasive pre-surgical assessment, endoscopic Ultrasound (EUS) is highly sensitive for local lymph node evaluation [4], while computed tomography (CT) and positron emission tomography (PET) scans are crucial for broader staging, including distant metastases [5]. Magnetic Resonance Imaging (MRI) may also be utilized, but less frequently for lymph node assessment [6]. These imaging techniques, though valuable for initial staging and planning, have limitations in detecting small LNM, particularly micrometastases, as demonstrated by the small median sizes of involved lymph nodes and metastatic nests in our study. The lower sensitivity of imaging modalities such as FDG-PET for detecting small LNMs highlights the challenge of relying solely on preoperative imaging for accurate nodal staging. Consequently, this underscores the need for meticulous surgical assessment and possibly more extensive lymph node dissection in certain cases, even when clinical staging suggests the absence of nodal involvement. The discrepancy between clinical and pathological findings emphasizes the potential for underestimation of disease spread and the critical role of postoperative pathological evaluation in guiding further treatment decisions and improving patient outcomes. Therefore, imaging methods cannot substitute the conclusive nature of histopathological examination post-surgery [7]. Histopathological samples are routinely obtained via surgical route, but surgery is no longer the preferred choice for treating metastatic esophageal cancer due to increased risks and poor prognosis, especially in patients with advanced metastases [8]. Therefore, imaging methods such as endoscopic ultrasound, CT, and 2-[fluorine-18]fluoro-2-deoxy-D-glucose (FDG)-PET for detecting LNM in esophageal cancer are critical since they are less invasive than surgery. Each of these modalities has its limitations, and individual studies suggest they exhibit low to moderate sensitivity and moderate to high specificity when assessing lymph node status [9]. The integration of artificial intelligence (AI) into radiology, propelled by advancements in machine learning (ML) and deep learning (DL), has ushered in a paradigm shift. This transformation is marked by the optimization of image acquisition processes, the streamlining of operational workflows, and the enhancement of diagnostic precision. ML algorithms, as evident in tools like Computer-Aided Diagnosis (CAD), significantly contribute to heightened sensitivity and specificity, reducing the time needed for interpreting chest X-rays. Deep learning models, particularly those built on convolutional neural networks (CNNs), demonstrate exceptional proficiency in tasks such as image recognition, proving invaluable for deciphering intricate medical images. Moreover, Radiomics plays a vital role in leveraging data to improve diagnostic insights by extracting quantitative features from medical images. This process is fundamental in enhancing our understanding of medical conditions through the analysis of specific image characteristics [10]. Radiomics, as an analytical method in medical imaging, employs sophisticated mathematical analyses to extract detailed features from medical images (e.g., CT, MRI, and PET), with a primary application focus on oncology [11]. Radiomics seeks to transform medical images into data that can be mined for valuable insights not easily visible to the naked eye. Through the analysis of quantitative features, it aims to offer extra details about the inherent biology, diversity, and traits of tissues and tumors. The extracted information holds potential for diverse medical applications, especially in oncology, serving diagnostic, prognostic, and predictive purposes [12]. Radiomics plays a potential role in improving the staging of esophageal cancer by analyzing texture features from imaging modalities. These features, including tumor heterogeneity and various measurements, offer additional insights beyond conventional staging methods [13]. Radiomics outperforms traditional radiological assessments conducted by radiologists in various aspects of cancer diagnosis and prognosis. It excels in tasks such as predicting tumor invasion and differentiating between malignant and benign tumors, offering promising potential for accurate prognosis and treatment planning. Radiomics models also show efficacy in forecasting metastasis, providing valuable insights for personalized patient care. These findings underscore radiomics’ role in improving diagnostic accuracy and guiding clinical decision-making in oncology [14]. Numerous meta-analyses have highlighted the promising results of the radiomics methods for predicting LNM in malignancies of organs such as the stomach [15], thyroid [16], breast [17], cervix [18], and pancreas [19]. The pooled area under the curve (AUC) of these studies fell between 0.70 and 0.90, indicating moderate to good diagnostic performance of radiomics methods for predicting LNM. Due to the variable diagnostic performance of radiomics methods for predicting LNM in different organs, it is necessary to obtain a comprehensive standpoint on the accuracy and quality of the radiomics studies in esophageal cancer. This objective can be achieved through a systematic approach and meta-analysis. Therfore, this study was designed to investigate the pooled diagnostic performance and quality of the published literature, as well as to provide future perspectives for further studies.

## Materials and methods

### Study design and reporting guidelines

The present study has been conducted following the Preferred Reporting Items for Systematic Reviews and Meta-analysis (PRISMA 2020) guidelines [20].

### Literature search

A systematic literature search of the electronic databases, including Embase, PubMed, and Web of Science, was performed independently by two reviewers to identify relevant studies that predicted lymph node metastasis in esophageal cancer using the following terms and their equivalents: (Radiomics) AND (Esophageal Cancer) AND (Lymph Node Metastasis). The search was updated on November 16, 2023. Exclusively, English-language studies were taken into account. The updated search terms and the results are detailed in Supplementary Materials.

### Study selection

PICO (population, intervention, comparison, and outcome) questions of the study were: (P) population: patients preoperatively diagnosed with esophageal cancer; (I) intervention: application of radiomics; (C) comparison: assessment of radiomics for prediction of LNM before treatment; and (O) outcome: measurement of diagnostic performance (e.g., sensitivity, specificity, and AUC) for predicting LNM after surgery. Inclusion criteria were (a) application of radiomics to predict LNM in esophageal cancer, (b) all participants had pathological postoperative LNM status (c) sufficient data for calculating 2 × 2 contingency tables consisting of true positive (TP), false positive (FP), false negative (FN), and true negative (TN). The exclusion criteria were as follows: (a) review papers, case reports, meetings, letters, abstracts, editorials, comments, posters, and guidelines; (b) studies that did not use radiomics methods for predicting LNM; (c) articles with no access; (d) literature published in a language other than English; (d) not providing enough data for constructing 2 × 2 tables; and (e) studies that not used separate validation cohorts.

### Data extraction

The citations obtained through database retrieval were imported into Endnote software. After removing redundant publications, a thorough examination of titles and abstracts was conducted to eliminate literature that did not meet the specified inclusion criteria. Following this, the complete texts of the remaining studies were carefully reviewed to ascertain the definitive inclusion of literature. Two authors independently carried out data extraction and the evaluation of study quality. The following basic data was extracted: name of the first author with the year of publication, study origin, design of the study (e.g., retrospective or prospective design), number of centers, number of participants in validation cohorts, reference standard, image modality, phase of imaging acquiring (for CT scan studies), radiomics approach (texture analysis, ML, or DL), and combined clinicopathological features. In addition, the following technical information was extracted: segmentation method (automatic vs. manual), region of interest (ROI) type (2D vs. 3D), software used for feature extraction, number of imaging features extracted/selected, type of imaging features extracted, modeling algorithm, features reduction algorithm, ICC evaluation, and type of cross-validation.

### Quality assessment

A modified version of the Quality Assessment of Diagnostic Accuracy Studies (QUADAS-2) tool was designed to investigate the quality of the included studies and questions for each section are detailed in Table S4 (Supplementary Materials) [21]. In addition, the Radiomics Quality Score (RQS) tool proposed by Lamblin et al. was used to evaluate the methodological quality of radiomics studies [22]. QUADAS-2 questions were implemented in the Review Manager software, and diagrams were drawn subsequently.

### Statistical data analysis

The accumulative values of sensitivity (SENS), specificity (SPEC), diagnostic odds ratio (DOR), positive likelihood ratio (PLR), negative likelihood ratio (NLR), and are under the curve (AUC), with their 95% confidence intervals (CIs) were generated. Utilizing the random effects model, we generated the summary receiver operating characteristic (SROC) curve and calculated the AUC to appraise the diagnostic efficacy of the aggregated studies. The AUC values were categorized as indicating low (0.5–0.7), fair (0.7–0.8), good (0.8–0.9), and excellent discriminatory power (> 0.9). Coupled forest plots were generated to show the pooled value for sensitivity and specificity. Cochran’s Q test and Higgins’ I^2^ statistic were calculated to estimate the heterogeneity among the studies included in this meta-analysis, with I^2^ values categorized as follows: 0 to 25% indicating very low heterogeneity, 25 to 50% indicating low heterogeneity, 50 to 75% indicating medium heterogeneity, and > 75% indicating high heterogeneity. We used Deek’s asymmetry test and its funnel plot to investigate publication bias. All p-values below 0.05 were considered to be significant. The statistical analyses in this study were conducted using Stata version 17.0 and meta-DiSc. Fagan plots were employed to evaluate clinical effectiveness by offering post-test probabilities of LNM while considering pre-test probabilities.

## Results

### Literature search

**Fig. 1:**
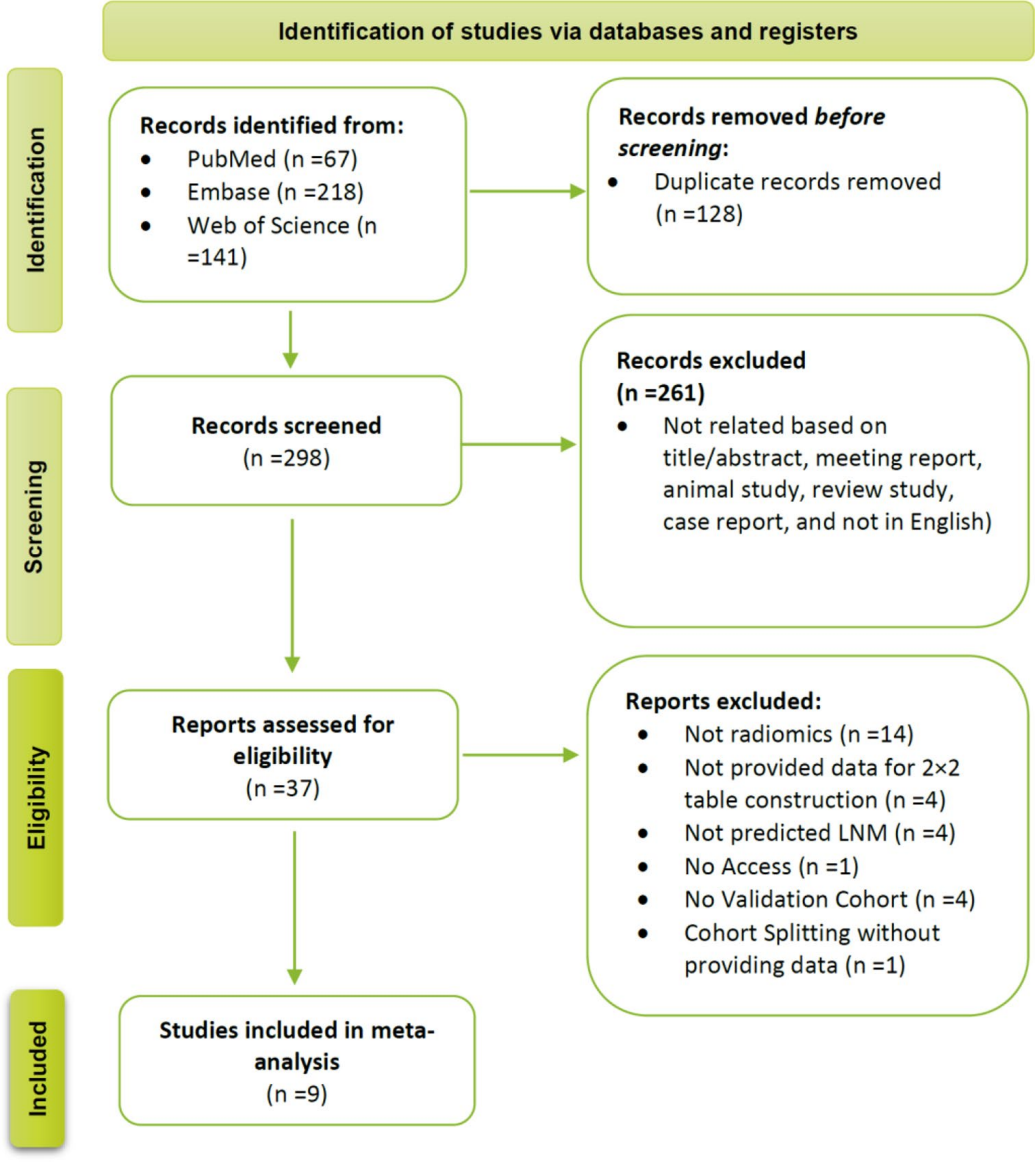
Flowchart of the study based on PRISMA guidelines

An electronic database search identified 426 citations with 128 duplicate studies. After screening the titles and abstracts of the candidate studies, 261 citations were excluded for not meeting the inclusion criteria. A thorough examination of the full texts resulted in the exclusion of 28 additional articles, leaving 9 for inclusion in the meta-analysis [23–31]. Figure 1 illustrates the detailed search process.

### Study characteristics

Table 1 shows full characteristics of the selected studies and predictive models. Nine articles consisting of 719 participants were selected in the quantitative synthesis, all retrospectively designed, eight conducted in China [23–26, 28–31] and one in the United Kingdom [20]. Imaging modalities were CT [23–26, 28–30], PET [27], and MRI [31]. Only one study used multi-center data [27]. Two studies used deep learning-based radiomics (deep-radiomics) for feature extraction [26, 28], and the rest of the studies were conventional (machine learning-based) radiomics [23–25, 27, 29–31]. Five studies combined radiomics and clinical features [26–30]. Manual ROI segmentation was performed by seven studies [23–26, 29–31], and only two studies [27, 28] used the automatic segmentation method. Only one study used 2D ROI segmentation [28]. Matlab was the most frequently used software for feature extraction (5/9) [24, 25, 27, 28, 31]. Similarly, the least absolute shrinkage and selection operator (LASSO) algorithm was adopted in two-thirds of the studies for feature selection [23, 25, 27–30], followed by more modern algorithms such as “elastic net” [24, 26, 31]. Logistic regression (LR) was the most commonly adopted algorithm for building radiomic models, and only one study used more advanced machine learning algorithms such as support vector machine (SVM), AdaBoost (Adaptive Boosting), and random forest (RF) [28].

### Quality assessment

#### QUADAS-2

**Fig. 2:**
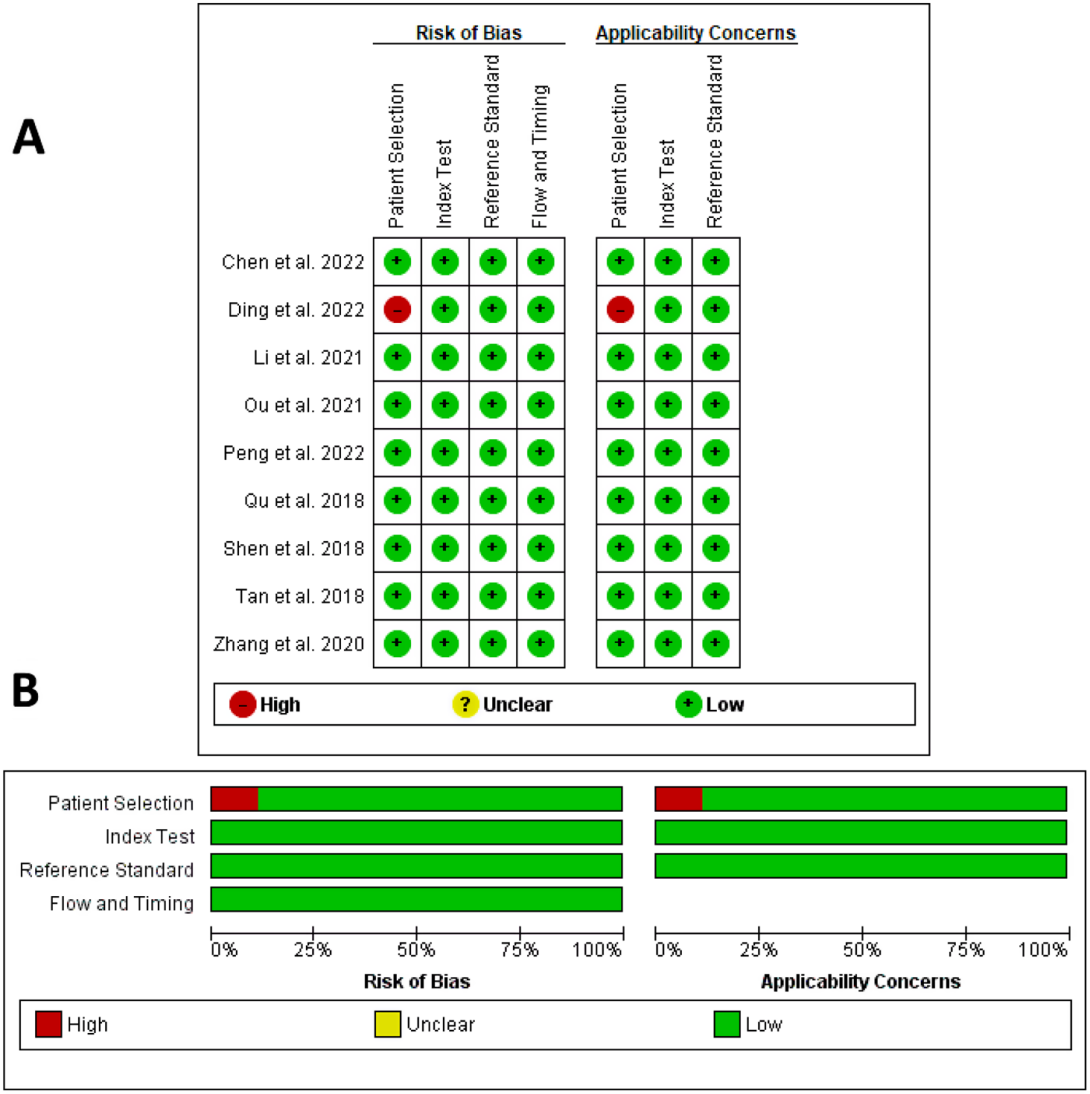
QUADAS quality assessment per study (**A**) and per domain (**B**)

Figure 2 shows the quality of the selected studies using QUADAS-2 tool, which was completely acceptable, and their design was aligned with the signaling questions. Only the study by Ding et al. had a high risk of bias and a high applicability concern in the patient selection domain as it included some patients who received treatment before imaging [26].

**Table 1:** Characteristics of the included studies and predictive models

First Author/Year of Publication	Country	Study design	Number of centers	Patients in validation cohort	Image Modality	Phase	Radiomics approach	Clinical features	Segmentation Method	ROI type (2D vs. 3D)	Feature extraction software	Selected features(n)/Extracted features (n	Type of features	Feature selection algorithm	ICC Evaluation	Radiomic Model Construction Algorithm
Li et al. 2021 [23]	China	Retro	Single	25+ 35-	CT	P + A + V	Machine Learning	No	Manual	3D	Wise Multimodal Research Platform	26/2107	First-order, shape, and texture features	ANOVA and LASSO	>0.8	LR
Shen et al. 2018 [24]	China	Retro	Single	19+ 38-	CT	A	Machine Learning	No	Manual	3D	Matlab	13/788	First-order histogram statistics, GLCM, GLRL, fractal dimensions, and wavelet filtered	elastic-net	0.873	LR
Ou et al. 2021 [25]	China	Retro	Single	60+ 41-	CT	A	Machine Learning	No	Manual	3D	Matlab	11/537	Shape, intensity histogram, GLCM, and GLRLM	LASSO	0.75	LR
Ding et al. 2022 [26]	China	Retro	Single	45+ 55-	CT	P + A + V	Deep Learning	Yes	Manual	3D	Pytorch	14/128	Equivalent diameter, extent, principal axis length, convex volume, solidity, surface area, volume, GLCM, GLRLM, GLSZM, and NGTDM.	feature-wise attentional graph neural network (FAGNN, 3D-UNet)	-	LR
Zhang et al. 2020 [27]	UK	Retro	Two	35+ 25-	PET	-	Machine Learning	Yes	Automatic	3D	MATLAB	9/154	-	RFE and LASSO	-	LR
Chen et al. 2022 [28]	China	Retro	Single	46+ 46-	CT	NM	Deep Learning	Yes	Automatic	2D	MATLAB and ResNet algorithm in Python	10/207 (handcrafted) and 40/1000 (deep radiomics)	GLCM, GLRLM, Gabor, HOG, and local entropy as textural features, and HU, Phase, and Hessian features as the shape features	T-test and LASSO	-	SVM, AdaBoost, and RF
Peng et al. 2022 [29]	China	Retro	Single	41+ 40-	CT	A+P	Machine Learning	Yes	Manual	3D	PyRadiomics	16 (CT) and 18 (CECT)/1502	First-order statistics, shape-based features (3D/2D), GLCM, GLSZM, GLRLM, NGTDM, GLDM, and high older features	LASSO	0.75	LR
Tan et al. 2018 [30]	China	Retro	Single	37+ 39-	CT	A	Machine Learning	Yes	Manual	3D	PyRadiomics	5/1576	First order, shape and texture features derived from GLCM, GLSZM, and GLRLM	LASSO	0.9	LR
Qu et al. 2018 [31]	China	Retro	Single	26+ 66-	MRI	-	Machine Learning	No	Manual	3D	MATLAB	9/1578	Shape, first-order histogram, texture, and wavelet group analysis	elastic net	0.943	LR

**Abbreviations**: Plain (P), Arterial Phase (A), Venous Phase (V), Magnetic Resonance Imaging (MRI), Analysis of Variance (ANOVA), Least Absolute Shrinkage and Selection Operator (LASSO), Support Vector Machine (SVM), AdaBoost (Adaptive Boosting), Random Forest (RF), Gray Level Co-occurrence Matrix (GLCM), Gray Level Run Length (GLRL), Gray Level Run Length Matrix (GLRLM), Gray Level Size Zone Matrix (GLSZM), Neighboring Gray Tone Difference Matrix (NGTDM), Histogram of Oriented Gradients (HOG), and Gray Level Dependence Matrix (GLDM)

#### RQS

The nine studies obtained an average RQS score of 12.78 and a median score of 12, with individual scores ranging from 9 to 14 out of 36 points. The estimated mean score was 35%, and the study with the superior rating achieved 38%. All studies provided/performed detailed image protocol quality, feature reduction, and discrimination statistics. On the other hand, none of the studies provided a phantom study, prospective study, biological correlation, comparison to the gold standard, cost-effectiveness analyses, and open science and data. Multiple segmentation was not performed in one study [30]. One-third of the studies did imaging at multiple points [23, 26, 29]. Multivariable analysis (combined with clinical factors) was performed in two-thirds of the studies [24, 26–30]. Likewise, cut-off analysis was performed only in two studies [25, 27]. Eight of the included studies had validation cohorts and received + 2 points in validation items, and one study [27], due to using data for another center, received + 3 points. Four studies assessed potential clinical applicability by conducting decision curve analysis [23, 24, 29, 30]. Detailed RQS scores of each study are provided in Table 2.

**Table 2:** RQS score of the included studies per item

Study	A	B	C	D	E	F	G	H	I	J	K	L	M	*N*	O	*P*	RQS
Shen et al. 2018	1	1	0	0	3	1	0	0	2	2	0	2	0	2	0	0	14
Li et al. 2021	1	1	0	1	3	0	0	0	2	0	0	2	0	2	0	0	12
Ou et al. 2021	1	1	0	0	3	0	0	1	2	0	0	2	0	0	0	0	10
Ding et al. 2022	1	1	0	1	3	1	0	0	2	0	0	2	0	0	0	0	11
Zhang et al. 2020	1	1	0	0	3	1	0	1	2	1	0	3	0	0	0	0	13
Chen et al. 2022	1	1	0	0	3	1	0	0	2	0	0	2	0	0	0	0	10
Peng et al. 2022	1	1	0	1	3	1	0	0	2	1	0	2	0	2	0	0	14
Tan et al. 2018	1	0	0	0	3	1	0	0	2	1	0	2	0	2	0	0	12
Qu et al. 2018	1	1	0	0	3	0	0	0	2	0	0	2	0	0	0	0	9
Average	1	0.89	0	0.33	3	0.67	0	0.22	2	0.55	0	2.11	0	0.89	0	0	**12.78**

Abbreviations: A: Image Protocol Quality, B: Multiple Segmentation, C: Phantom Study, D: Imaging at Multiple Points, E: Feature Reduction, F: Multivariable Analyses, G: Biological Correlation, H: Cut-off Analyses, I: Discrimination Statics, J: Calibration Statics, K: Prospective Study, L: Validation, M: Comparison to Gold Standard, N: Potential Clinical Application, O: Cost Effectiveness Analyses, P: Open science and Data

### Diagnostic meta-analysis

**Fig. 3:**
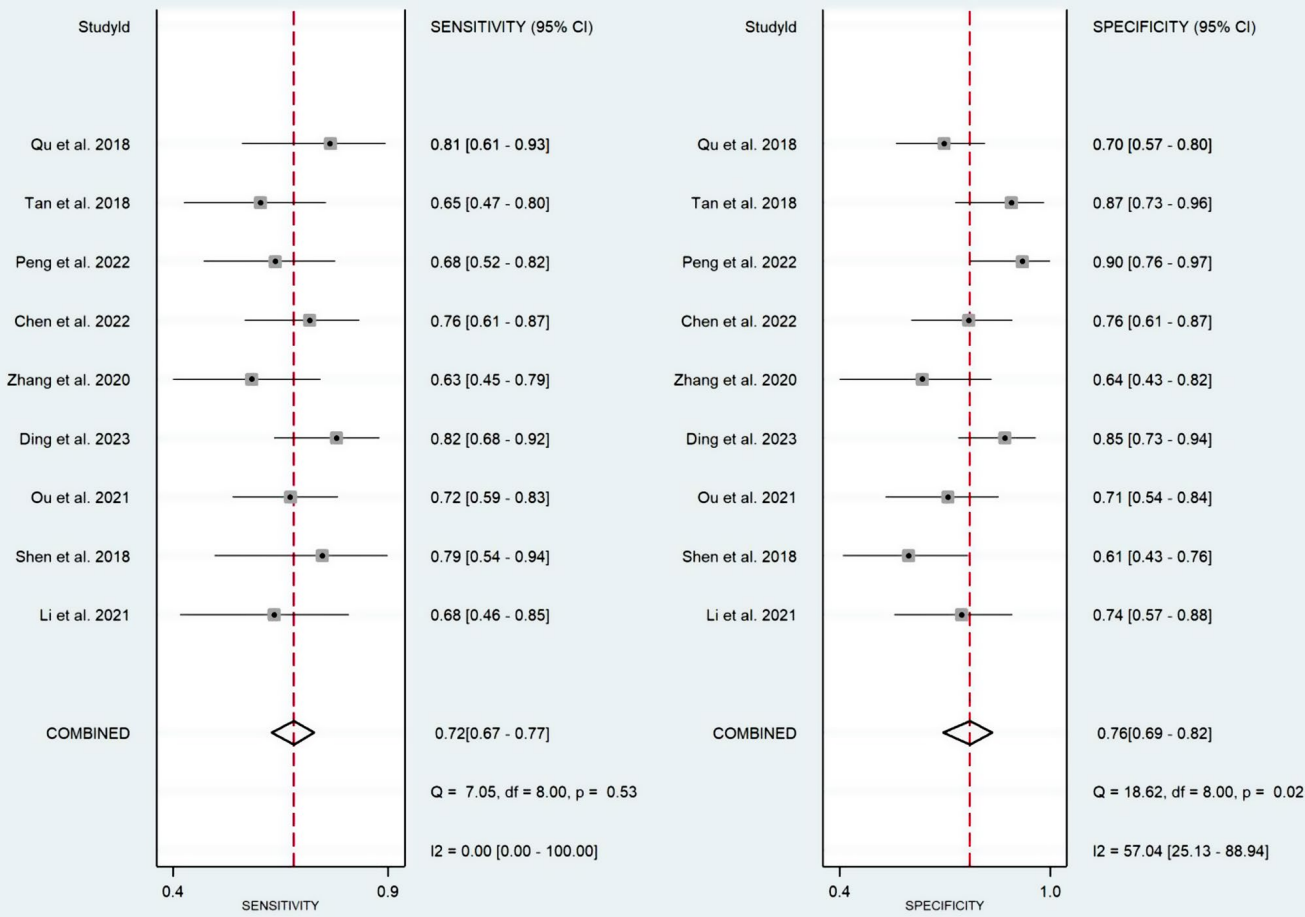
Coupled forest plot showing pooled sensitivity and specificity

**Fig. 4:**
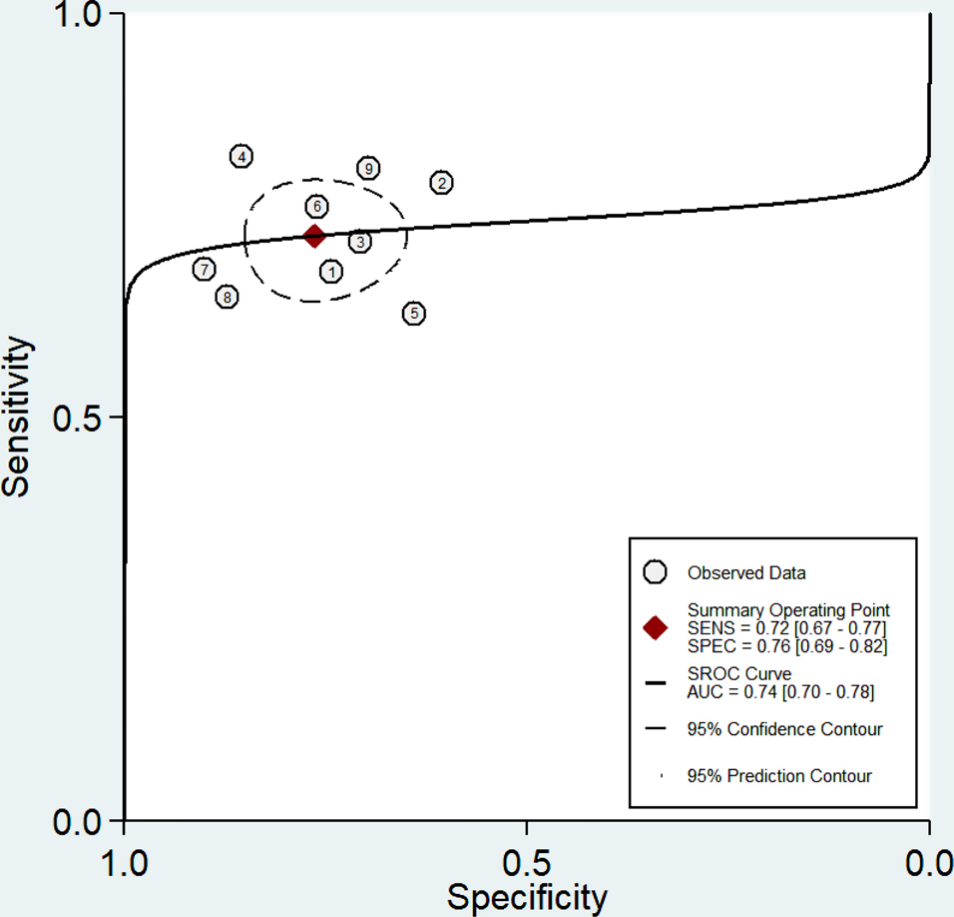
Summary ROC curve (SROC) of the radiomic models for predicting LNM in esophageal cancer

Nine studies (validation cohorts) consisting of 334 patients with LNM (+) and 385 patients without LNM (-) were selected for the quantitative synthesis. The pooled diagnostic indicators with their 95% confidence interval (CI) were determined: SENS, 0.72 [95% CI; 0.67–0.77]; SPEC, 0.76 [95% CI; 0.69–0.82]; PLR, 3.1 [95% CI; 2.3–4.1]; NLR, 0.36 [95% CI; 0.30–0.44]; DOR, 9 [95% CI; 6–13]; and AUC, 0.74 [95% CI; 0.70–0.78]. The coupled forest plot, including sensitivity and specificity alongside heterogeneity indicators (Higgins’ I^2^ and Cochran’s Q) is shown in Fig. 3. Furthermore, Fig. 4 shows the summary ROC curve (SROC) with pooled AUC value.

### Heterogeneity

#### Heterogeneity existence

The Cochran’s Q and Higgins I^2^ tests showed that medium heterogeneity (I^2^ = 57.04%) was present in the pooled specificity values (**p-value = 0.02**). In contrast, very low heterogeneity (I^2^ = 0.00%) was observed in the accumulative sensitivity, and Cochran’s Q test did not show a significant heterogeneity (p-value = 0.53). Threshold effect was also ruled out as the possible cause of heterogeneity since Spearman’s correlation coefficient (r) was calculated as 0.1 (p-value = 0.798).

#### Causes of heterogeneity

Meta-regression was performed to investigate the causes of heterogeneity (Table 3). However, among all of the considered covariates, only using combined models significantly contributed to the results’ heterogeneity (**p-value = 0.05**). Among other covariates, a sample size higher than 75 or using least absolute shrinkage and selection operator (LASSO) for feature extraction might be implicated in the inter-study heterogeneity. However, the results were not statistically significant (0.05 < p-value < 0.10).

### Subgroup analysis

Different factors were considered for subgroup analysis (Table 3).

#### Study population

Studies with sample sizes larger than 75 showed higher pooled sensitivity (0.74 vs. 0.68) and pooled specificity (0.80 vs. 0.67); however, the results were not statistically significant (p-values for sensitivity and specificity > 0.05).

#### Publication year

Studies published before 2020 exhibited slightly higher pooled sensitivity (0.73 vs. 0.72; p-value = 0.01), but pooled specificity was higher in those published after 2020 (0.78 vs. 0.71; p-value > 0.05 with no statistically significant difference).

#### ROI segmentation method

Studies utilizing manual ROI segmentation exhibited higher pooled sensitivity (0.73 vs. 0.70; p-value = 0.04) and pooled specificity (0.78 vs. 0.71; p-value = 0.38, not statistically significant) compared to those with semi-automatic segmentation.

#### ROI segmentation dimension

Studies utilizing 2D ROI segmentation demonstrated superior sensitivity (0.76 vs. 0.72; p-value = 0.03) compared to 3D segmentation; however, specificity was quite similar (0.76; p-value = 0.35).

#### Radiomics methods

Deep learning-based radiomics exhibited superior sensitivity (0.79 vs. 0.70) and specificity (0.81 vs. 0.75) compared to conventional radiomics methods, but the evidence was not statistically significant (p-value > 0.05) due to the small number of studies employing the deep learning approach (*n* = 2).

#### Imaging modality

MRI demonstrated the highest sensitivity value (0.81), followed by CT (0.73) and PET (0.63). As for specificity, CT scored the highest (0.79), followed by MRI (0.70) and PET (0.64). Notably, due to the limited number of studies involving MRI or PET, the results lacked statistical significance (p-value > 0.05), underscoring the need for further investigation into MRI and PET radiomics in this area.

#### Radiomics model construction algorithm

A study that used AdaBoost for model construction had a significantly higher sensitivity compared to those other with LR (0.76 vs. 0.72; p-value = 0.03). However, the specificity was pretty similar (0.76; p-value = 0.35).

#### Feature selection algorithm

Studies employing Elastic Net feature selection exhibited significantly higher sensitivity (0.81 vs. 0.69; p-value = 0.00). However, the pooled specificity was higher for studies utilizing the LASSO algorithm (0.78 vs. 0.73; p-value = 0.13), although this difference lacked statistically significant evidence.

#### Combined radiomics models

Studies combining radiomics signature with clinical factors demonstrated a significantly lower pooled sensitivity compared to those utilizing signature-only studies (0.74 vs. 0.72). In contrast, combined models exhibited a higher pooled specificity (0.82 vs. 0.69; p-value = 0.05).

**Table 3:** Meta-regression and subgroup analysis based on different covariates

Subgroups (Factors)		N	SENS	P_SEN_	SPEC	P_SPEC_	Meta regression	
							Likelihood ratio (chi^2^)	P-value
Number of patients	> 75	6	0.74 [0.68–0.79]	0.10	0.80 [0.74–0.86]	0.45	5.08	0.08
	< 75	3	0.68 [0.58–0.79]		0.67 [0.55–0.78]			
Segmentation	Manual	7	0.73 [0.68–0.79]	0.04	0.78 [0.71–0.85]	0.38	0.84	0.66
	Semiautomatic	2	0.70 [0.60–0.80]		0.71 [0.56–0.87]			
Publication Year	After 2020	6	0.72 [0.67–0.78]	0.01	0.78 [0.71–0.85]	0.13	0.62	0.73
	Before 2020	3	0.73 [0.64–0.83]		0.73 [0.62–0.84]			
ROI	3D	8	0.72 [0.67–0.77]	0.03	0.76 [0.70–0.83]	0.35	0.36	0.84
	2D	1	0.76 [0.64–0.88]		0.76 [0.58–0.95]			
Radiomics Method	Deep learning	2	0.79 [0.71–0.88]	0.13	0.81 [0.71–0.92]	0.23	4.00	0.14
	Conventional	7	0.70 [0.64–0.76]		0.75 [0.67–0.82]			
Imaging Modality	PET	1	0.63 [0.46–0.79]	0.03	0.64 [0.39–0.89]	0.13	3.16	0.21
	Others	8	0.74 [0.69–0.79]		0.77 [0.71–0.84]			
Imaging Modality	MRI	1	0.81 [0.66–0.96]	0.71	0.70 [0.51–0.88]	0.10	1.64	0.44
	Others	8	0.72 [0.67–0.77]		0.77 [0.71–0.84]			
Imaging Modality	CT	7	0.73 [0.68–0.78]	0.07	0.79 [0.72–0.85]	0.50	2.14	0.34
	Others	2	0.70 [0.59–0.82]		0.68 [0.54–0.82]			
Model Construction Algorithm	LR	8	0.72 [0.67–0.77]	0.03	0.76 [0.70–0.83]	0.35	0.36	0.84
	AdaBoost	1	0.76 [0.64–0.88]		0.76 [0.58–0.95]			
Feature Selection Algorithm	LASSO	6	0.69 [0.63–0.75]	0.00	0.78 [0.71–0.86]	0.13	5.44	0.07
	Elastic Net	3	0.81 [0.73–0.89]		0.73 [0.62–0.84]			
Combined Clinical Factors	Yes	5	0.72 [0.65–0.78]	0.00	0.82 [0.77–0.87]	0.05	6.02	**0.05**
	No	4	0.74 [0.66–0.81]		0.69 [0.62–0.76]			

### Publication bias

**Fig. 5:**
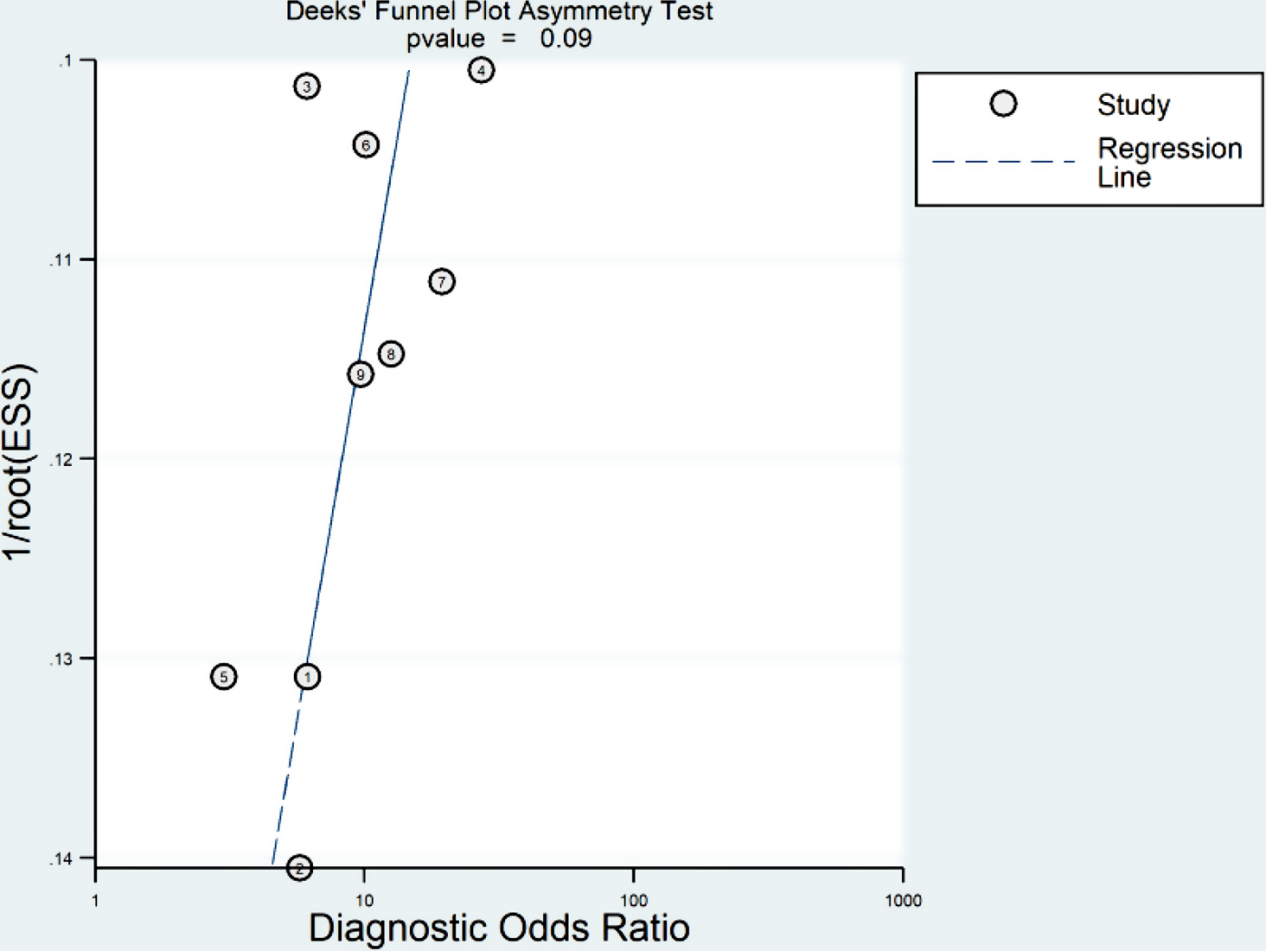
Deeks’ funnel plot for testing publication bias (p-value = 0.09)

No significant publication bias was found in the included studies using Deeks’ asymmetry test (p-value = 0.09) (Fig. 5).

### Sensitivity analysis

By removing each study one by one, the pooled AUC varied between 0.73 and 0.78, with the latter belonging to the removal of the study by Zhang et al., which used PET radiomics (Table 4). Overall, the pooled values were almost consistent, indicating the robustness of the results.

**Table 4:** Results of the sensitivity analysis

Study Removed	SEN	SPEC	PLR	NLR	DOR	AUC
Chen et al. 2022	0.72	0.75	3.1	0.37	8	0.73
Ding et al. 2023	0.71	0.75	2.8	0.39	7	0.76
Li et al. 2021	0.73	0.77	3.1	0.35	9	0.74
Ou et al. 2021	0.73	0.77	3.2	0.35	9	0.74
Peng et al. 2022	0.73	0.74	2.8	0.3	8	0.76
Qu et al. 2018	0.72	0.77	3.1	0.37	9	0.73
Shen et al. 2018	0.72	0.78	3.3	0.36	9	0.74
Tan et al. 2018	0.73	0.75	2.9	0.36	0.36	0.76
Zhang et al. 2020	0.74	0.78	3.3	0.34	10	0.78

### Clinical utility

**Fig. 6:**
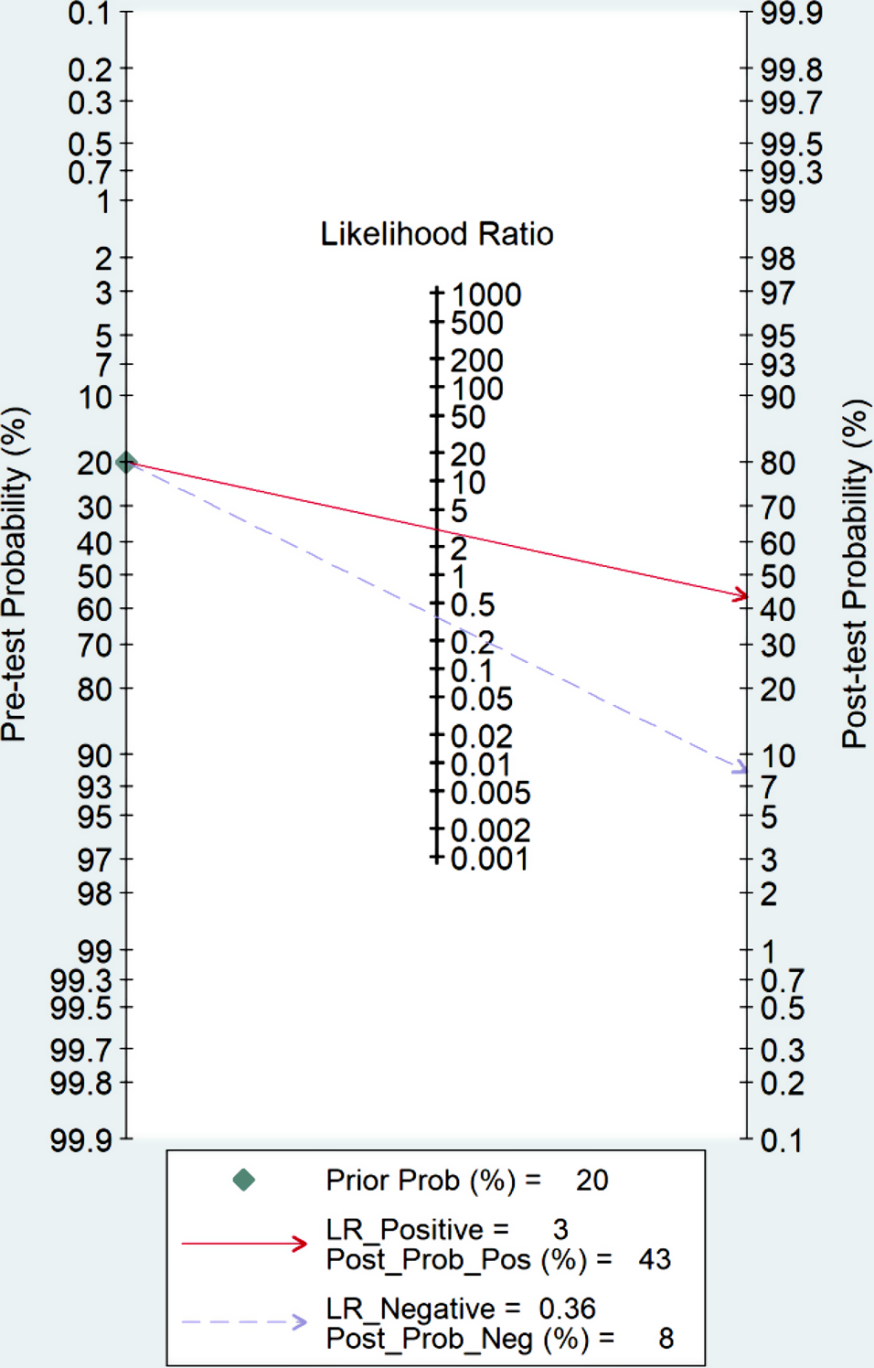
Fagan plot showing the clinical utility of radiomics models for predicting LNM in esophageal cancer

Utilizing radiomics models resulted in a rise in the post-test probability from 20 to 43% when the initial probability was positive, accompanied by a positive likelihood ratio of 3. Conversely, when the initial probability was negative, the post-test probability diminished to 8%, featuring a negative likelihood ratio of 0.36 (Fig. 6).

## Discussion

Lymph node metastasis plays a crucial role in esophageal cancer prognosis, particularly impacting early-stage disease due to the anatomical and histological characteristics of esophageal cancer [32]. Esophageal cancer is recognized for its aggressive behavior and frequent lymphatic dissemination, underscoring the pivotal role of lymph node status as a critical factor in predicting patient outcomes. Achieving precise preoperative staging is imperative for informed decision-making and effective management of esophageal cancer. Despite the widespread use of esophageal CT scans in preoperative assessments, their reliability in detecting lymph node (LN) involvement is deemed inadequate. This inadequacy is attributed to disagreements in diagnostic criteria and inherent limitations, including the challenge of identifying metastasis that may not result in noticeable enlargement of the lymph nodes [33]. Although large-scale lymph node (LN) dissection is necessary during surgery, excessive LN dissection is associated with postoperative complications. Therefore, accurate preoperative prediction of LNM can prevent unnecessary lymph node dissection [26]. Recent advances in artificial intelligence in imaging, particularly radiomics, opened up a new horizon in precision medicine [34, 35]. The results of the meta-analysis consisting of nine studies with separate validation cohorts and acceptable overall quality showed that radiomics-based methods have a moderate diagnostic performance (AUC = 0.74) for diagnosing LNM in esophageal cancer. The presence of a geographic bias, with the majority of studies (8 out of 9) originating from China, raises a concern about the representativeness of the evidence. This concentration may introduce regional variations that limit the generalizability of findings to a broader global context. The disproportionate focus on a specific geographic region underscores the importance of diversifying study locations to capture a more comprehensive understanding of the subject matter. Future research should strive for a more globally representative sample to ensure the applicability of findings across different populations and settings. In addition, The retrospective study design in the included studies is a limitation, as it poses challenges related to data accuracy, potential biases, and establishing causal relationships. Retrospective studies lack prospective data collection and may have incomplete variables. Despite providing insights, their design introduces limitations that should be considered when interpreting findings. Future research could improve validity by incorporating prospective study designs.

Compared to previous meta-analyses in other gastrointestinal cancers, the pooled diagnostic performance was slightly lower in our study. In rectal cancer, a meta-analysis by Bedrikovetski et al. showed that the pooled AUC of radiomics models was 0.808, which is higher than the results of this study [28]. A recently published meta-analysis showed that CT-scan-based radiomics combined with clinical factors could reach an AUC of 0.90, representing excellent diagnostic accuracy [15]. Another meta-analysis evaluating validation cohorts has shown that radiomics based on MRI and CT might facilitate the diagnosis of LNM in pancreatic ductal adenocarcinoma with a pooled AUC of 0.79 [19]. However, it seems that radiomics methods might perform slightly weaker in thoracic and head and neck regions compared to the abdominal cavity, as another meta-analysis has shown that CT-based radiomics studies have a pooled AUC of 0.75 for predicting LNM in thyroid cancer [16]. This suggests that the current performance of radiomics studies falls within a fair range of diagnostic accuracy. Such findings highlight the necessity for more refined methodologies and enhanced study designs to improve the diagnostic capabilities of radiomics in identifying LNM in esophageal cancer. Future research should prioritize the standardization of imaging protocols, feature extraction methods, and deep learning algorithms. Additionally, to ensure their generalizability across different populations and clinical settings, it is crucial to train and validate these models using larger and more diverse external datasets.

We concluded following findings based on subgroup analysis: First, it seems that 2D segmentation performs better, at least in terms of sensitivity, compared to the 3D segmentation method, as this finding was previously mentioned in meta-analyses of thyroid and gastric cancers [15, 16]. This observation can be attributed to several factors: First, 2D images often offer higher resolution and quality within specific planes, facilitating the detection of subtle features indicative of early disease. The simplicity and focused nature of 2D segmentation enable more precise analysis of certain anatomical features, while the computational efficiency of 2D methods allows for greater optimization during algorithm training. Additionally, the wider availability of annotated 2D data enhances the development of sensitive detection models. Despite the comprehensive spatial insights provided by 3D segmentation, its complexity may hinder the accurate modeling of early-stage disease markers. The choice between 2D and 3D approaches should, therefore, consider the specific clinical needs, the disease in question, and the goals of the imaging analysis [36]. The scarcity of studies employing 2D segmentation may result in inaccurate conclusions, restricting comprehensive insights and generalizability in this specific area. This constraint hampers a thorough exploration of potential applications and biases associated with 2D segmentation. To address this, future research should prioritize expanding the number of studies utilizing 2D segmentation to enhance understanding and assessment of its capabilities and limitations.

We also found that manual segmentation outperforms automatic segmentation in terms of sensitivity. However, it should be noted only one study used automatic segmentation, and further investigations are required in this context, as a previous meta-analysis mentioned the superiority of automatic segmentation [15]. The scarcity of studies utilizing automatic segmentation limits available evidence and constrains insights and generalizability in this area. This constraint impedes thorough exploration of potential applications and biases. Future research should prioritize expanding studies employing automatic segmentation to enhance understanding of its capabilities and limitations.

In addition, the pooled AUC of deep radiomics models was higher than the conventional models. However, the variations were not identified as statistically significant because of the limited number of studies examining this aspect (2 out of 9). The integration of CNNs and deep learning into radiomics has markedly enhanced diagnostic accuracy in medical imaging by automating the extraction of intricate features that may not be visible to the human eye. This advancement allows for the handling of high-dimensional data and the extraction of meaningful patterns, leading to improved disease detection, classification, and prediction capabilities. As these models are trained on large datasets, their diagnostic precision improves, offering potential for personalized medicine through predictive modeling of disease progression and treatment outcomes. Despite challenges such as the need for extensive annotated datasets, potential biases, and the complexity of interpreting deep learning models, this integration represents a significant leap forward in the field of medical imaging, promising more accurate, efficient, and individualized patient care [37–41]. Going forward, it’s crucial to increase the number of deep radiomics studies to get more comprehensive insights and facilitate thorough analyses and meta-analyses.

We also observed that adding clinical factors to radiomics signature can also be considered as a promising method to increase the diagnostic accuracy of the studies. Incorporating clinical factors into radiomics signatures enhances diagnostic accuracy by leveraging a comprehensive patient profile that combines macroscopic clinical data with microscopic imaging features. This integration improves specificity and sensitivity by helping differentiate diseases with similar imaging appearances and supports personalized medicine by accounting for individual variability in disease presentation. Additionally, it aids in accurate risk stratification, allowing for tailored treatment strategies and closer patient monitoring. The approach also enhances the generalizability of models across different populations by incorporating a wider range of predictive variables. Furthermore, aligning radiomics with established clinical practices bolsters the credibility and acceptance of these advanced diagnostic tools within the medical community, ensuring a smoother integration into clinical workflows. The synergy between clinical factors and radiomics signatures thus represents a significant step forward in developing more accurate, personalized, and clinically relevant diagnostic methodologies [42].

We have also shown that PET radiomcis methods are not superior to CT and MRI models, and comparing their performance with CT-scan methods requires more studies to establish a firm conclusion. The limitation of a limited number of studies utilizing the MRI and PET imaging modality was evident, with the majority (7 out of 9) relying on CT, one on PET, and only one incorporating MRI. This imbalance raises concerns about the comprehensiveness of insights gained from MRI and PET in the context of the topic under investigation. Considering the potential superiority of MRI in terms of performance [43–45], it emphasizes the crucial need for more extensive evaluation of its diagnostic accuracy in future research. This would ensure a comprehensive understanding of the subject matter and provide insights into the comparative effectiveness of different imaging modalities.

In radiomics model construction algorithms, we observed that AdaBoost had a significantly higher sensitivity compared to those studies using LR. A recent meta-analysis suggests that using more advanced machine learning algorithms such as support vector machines and AdaBosst can improve the results significantly, supported by our results [21]. AdaBoost, a machine learning algorithm that combines multiple weak classifiers to form a strong classifier, has shown significantly higher sensitivity in detecting specific conditions or characteristics from medical images compared to LR, a more traditional method widely applied in radiomics studies. This difference in performance can be attributed to AdaBoost’s ability to adaptively focus on the most challenging cases in the training dataset, thereby improving its ability to generalize from complex, high-dimensional imaging data. In contrast, LR, although powerful in its simplicity and interpretability, might struggle with the complex and high-dimensional nature of radiomic data. This adaptability of AdaBoost, coupled with its ability to handle a wide range of data distributions and its robustness to overfitting, likely contributes to its superior performance in sensitivity, as supported by both recent meta-analyses and empirical results [46, 47].

Regarding feature selection, we found that elastic net and feature-wise attentional graph neural networks might perform better than LASSO. Both elastic net and LASSO are regularization techniques used in linear regression, but while LASSO imposes variable sparsity by encouraging some coefficients to be exactly zero, elastic net combines both lasso and ridge regression penalties to provide a more balanced selection of variables [48, 49].

In this study, to compare the results of our study with previous meta-analyses, the overall quality of the selected articles was assessed using RQS tools, which is commonly used in systematic reviews for quality assessment of radiomics studies. Overall, the included studies received a mean score of 12.78, denoting 35% of the total possible score. This score is in line with the results of previous meta-analyses [15, 50], indicating that the included studies had an acceptable quality, and these results were also concluded from the QUADAS-2 assessment. However, following the development of new quality assessment tools for artificial intelligence like CLEAR and METRICS after 2023, we strongly recommend adopting these newer tools instead of RQS in future radiomics meta-analyses. The CLEAR checklist, short for Consolidated Criteria for Reporting Radiomics Studies, serves as a structured set of recommendations aimed at enhancing the transparency and quality of reporting in radiomics research. It stresses the importance of thorough documentation throughout every phase of a study, spanning from data collection and image processing to feature extraction and statistical analysis. What sets CLEAR apart from RQS is its broader focus on reporting standards rather than solely on methodological quality. By advocating for the transparent sharing of data, scripts, and models, CLEAR addresses the crucial need for reproducibility and validation in radiomics. Additionally, it offers specific guidance on how to report the workflow of radiomics studies, which is often overlooked. This holistic approach not only facilitates comparison, replication, and expansion of radiomics research but also aims to bolster the credibility and impact of findings within the field. On the other hand, METRICS (METhodological RadiomICs Score) is a novel scoring tool designed to assess the methodological quality of radiomics research, developed through a collaborative effort involving a large international panel of experts. Unlike existing tools such as the RQS, METRICS offers several advantages. Firstly, it incorporates input from a diverse group of experts through a modified Delphi process, ensuring a comprehensive and consensus-driven approach to evaluating research quality. Secondly, METRICS assigns weights to different categories and items based on expert rankings, providing a nuanced and transparent assessment framework. Thirdly, METRICS covers a wide range of methodological variations, including both traditional radiomics and deep learning-based approaches, making it applicable to diverse research contexts. Finally, METRICS is accompanied by a user-friendly web application and a repository for community feedback, facilitating its adoption and continuous improvement. Overall, METRICS represents a significant advancement in the field, offering a robust and adaptable tool for enhancing the methodological rigor of radiomics research [51, 52].

While high risk of bias for reference standard domain of one study was identified, it does not significantly compromise the overall reliability of our meta-analysis findings. The QUADAS-2 assessment tool was applied rigorously, and the majority of included studies demonstrated acceptable quality across the assessed domains. High risk of bias concerns, especially in diagnostic accuracy studies, are not uncommon, and variations in study design can contribute to these biases. Importantly, similar meta-analyses often encounter multiple instances of high risk of bias across various domains, making the presence of only one study with a high risk of bias in a single domain relatively favorable [21, 53, 54].

However, a medium to moderate degree of heterogeneity was observed based on Higgins’ I^2^ test for the pooled specificity. Following meta-regression, we found that integrating clinical factors with radiomics signatures might explain the possible cause of interstudy heterogeneity, as the diagnostic performance of combined models was higher. The pooled results were consistent regarding pooled sensitivity, and Higgins’ I^2^ test did not detect significant heterogeneity. In addition, no significant publication bias was observed based on Deek’s test. If no significant publication bias exists in a diagnostic test accuracy meta-analysis, it means that studies with positive and negative results are equally likely to be published. This leads to more representative and reliable findings, reduces the risk of overestimating the test’s accuracy, and allows for better-informed clinical decisions with improved generalizability across different populations and settings.

Although the pooled AUC in this study was 0.74, following removing a study that used PET-based radiomics (Zhang et al.) [27], we observed that the overall pooled AUC of the remaining studies (consisting of MRI and CT-scan modalities) increased to 0.78, proposing that CT or MR-based radiomics could improve the diagnostic performance.

## Limitations

This study faced a few limitations: First, commitment to methodological rigor drove the exclusion of studies lacking separate validation cohorts from the meta-analysis. Studies relying solely on training cohorts or cross-validation may lead to overestimating diagnostic accuracy, introducing a risk of overfitting and limiting the generalizability of results. The decision underscores the importance of assessing diagnostic models in independent datasets to ensure their applicability across diverse patient populations and clinical settings. Additionally, the study employed the RQS tool to assess the risk of bias, enhancing comparability with other studies. However, we recommend that future researchers consider utilizing newer tools such as METRICS and CLEAR for more comprehensive assessments. Moreover, while extracting data, we opted for the model exhibiting superior diagnostic efficacy from various options, potentially leading to an overestimation of the combined sensitivity and specificity of radiomics in LNM in esophageal cancer. Excluding studies published in Chinese could introduce bias by omitting potentially relevant data and perspectives, particularly from regions like China with significant research output. This exclusion may skew the overall understanding of the topic and introduce publication bias, as studies with statistically significant results may be more likely to be published in English-language journals. Therefore, researchers should carefully consider the implications of excluding studies based on language criteria to ensure the robustness and generalizability of their findings. Finally, it is important to acknowledge the limitation of pooling all imaging modalities together, including MRI, PET, and CT, in our study. While this approach allows for a comprehensive assessment of radiomics across various imaging techniques, it can also introduce heterogeneity in the data due to differences in image acquisition protocols, resolution, and contrast. However, the subgroup analysis was performed to rule out the possible sources of heterogeneity.

## Conclusion

This meta-analysis consolidates evidence on radiomics for predicting LNM in esophageal cancer, showcasing its potential diagnostic value. Despite identified heterogeneity and specific challenges, radiomics demonstrates promise in enhancing esophageal cancer staging. To integrate radiomics-based predictions into clinical workflows for esophageal cancer management, it is imperative to prioritize further research and development efforts aimed at refining radiomics models tailored to esophageal cancer while advocating for standardized imaging protocols and data-sharing initiatives. Validation through external testing using diverse datasets is essential to ensure the reliability and generalizability of radiomics models. Establishing guidelines for integration into clinical practice, developing decision support tools, and interdisciplinary collaboration between radiologists, oncologists, and surgeons are crucial steps. Prospective clinical trials are needed to evaluate the impact of radiomics on patient outcomes, and continuous evaluation and improvement of radiomics models are essential to keep pace with technological advancements and clinical needs. It’s noteworthy that when diagnostic performance reaches a level comparable to the gold standard, which is surgery for prediction of LNM in esophageal cancer, radiomics has the potential to replace it. However, we acknowledge that we are not currently at that stage, and further studies are required to achieve this level of performance.

### Electronic supplementary material

Below is the link to the electronic supplementary material.

Supplementary Material 1

## Data Availability

The original contributions presented in the study are included in the article. Further inquiries can be directed to the corresponding author.

## References

[1] Sung H, Ferlay J, Siegel RL, et al. Global cancer statistics 2020: GLOBOCAN estimates of incidence and mortality worldwide for 36 cancers in 185 countries. CA Cancer J Clin. 2021;71:209–49. 33538338 10.3322/caac.21660

[2] Hagens ERC, van Berge Henegouwen MI, Gisbertz SS. Distribution of lymph node metastases in esophageal carcinoma patients undergoing upfront surgery: a systematic review. Cancers (Basel). 2020;12:1592. 32560226 10.3390/cancers12061592 PMC7352338

[3] Kang H, Yang M, Zhang F et al. Identification lymph node metastasis in esophageal squamous cell carcinoma using whole slide images and a hybrid network of multiple instance and transfer learning. Biomed Signal Process Control. 2023; 82.

[4] Leng X-F, Zhu Y, Wang G-P, et al. Accuracy of ultrasound for the diagnosis of cervical lymph node metastasis in esophageal cancer: a systematic review and metaanalysis. J Thorac Dis. 2016;8:2146–57. 27621871 10.21037/jtd.2016.07.71 PMC4999759

[5] Chu L, Liu S, Guo T et al. Is performance of Fluorine-18-fluorodeoxyglucose Positron Emission Tomography/Computed tomography (CT) or contrast-enhanced CT efficient enough to Guide the Hilar Lymph Node Staging for Patients with esophageal squamous cell carcinoma? Front Oncol. 2022; 12. 10.3389/fonc.2022.814238 PMC8914423 35280825

[6] Shuto K, Kono T, Shiratori T, et al. Diagnostic performance of diffusion-weighted magnetic resonance imaging in assessing lymph node metastasis of esophageal cancer compared with PET. Esophagus. 2020;17:239–49. 31820208 10.1007/s10388-019-00704-w PMC7316698

[7] Aoyama J, Kawakubo H, Mayanagi S, et al. Discrepancy between the clinical and final pathological findings of lymph node metastasis in superficial esophageal cancer. Ann Surg Oncol. 2019;26:2874–81. 31209674 10.1245/s10434-019-07498-2

[8] Wang Y, Yang W, Wang Q, Zhou Y. Mechanisms of esophageal cancer metastasis and treatment progress. Front Immunol. 2023;14:1206504. 37359527 10.3389/fimmu.2023.1206504 PMC10285156

[9] Jayaprakasam VS, Yeh R, Ku GY, et al. Role of imaging in esophageal cancer management in 2020: update for radiologists. Am J Roentgenol. 2020;215:1072–84. 32901568 10.2214/AJR.20.22791

[10] Najjar R, Redefining Radiology. A review of Artificial Intelligence Integration in Medical Imaging. Diagnostics. 2023;13:2760. 37685300 10.3390/diagnostics13172760 PMC10487271

[11] Van Timmeren JE, Cester D, Tanadini-Lang S, et al. Radiomics in medical imaging—how-to guide and critical reflection. Insights Imaging. 2020;11:1–16. 32785796 10.1186/s13244-020-00887-2 PMC7423816

[12] Vernuccio F, Cannella R, Comelli A, et al. Radiomics and artificial intelligence: new frontiers in medicine. Recenti Prog Med. 2020;111:130–5. 32157259 10.1701/3315.32853

[13] van Rossum PSN, Xu C, Fried DV, et al. The emerging field of radiomics in esophageal cancer: current evidence and future potential. Transl Cancer Res. 2016;5:410. 30687593 10.21037/tcr.2016.06.19 PMC6343849

[14] Tramanzoli P, Castellani D, De Stefano V, et al. Radiomics vs radiologist in bladder and renal cancer. Results from a systematic review. Cent Eur J Urol. 2023;76:12. 10.5173/ceju.2023.252 PMC10091893 37064257

[15] HajiEsmailPoor Z, Tabnak P, Baradaran B et al. Diagnostic performance of CT-Scan based Radiomics for Prediction of Lymph Node Metastasis in Gastric Cancer: a systematic review and Meta-analysis. Front Oncol 13: 1185663. 10.3389/fonc.2023.1185663 PMC10627242 37936604

[16] HajiEsmailPoor Z, Kargar Z, Tabnak P. Radiomics diagnostic performance in predicting lymph node metastasis of papillary thyroid carcinoma: a systematic review and meta-analysis. Eur J Radiol. 2023; 111129. 10.1016/j.ejrad.2023.111129 37820522

[17] Gong X, Guo Y, Zhu T et al. Diagnostic performance of radiomics in predicting axillary lymph node metastasis in breast cancer: a systematic review and meta-analysis. Front Oncol. 2022; 12. 10.3389/fonc.2022.1046005 PMC9742555 36518318

[18] Ren J, Li Y, Liu X-Y et al. Diagnostic performance of ADC values and MRI-based radiomics analysis for detecting lymph node metastasis in patients with cervical cancer: a systematic review and meta-analysis. Eur J Radiol. 2022; 110504. 10.1016/j.ejrad.2022.110504 36108474

[19] Mirza-Aghazadeh-Attari M, Madani SP, Shahbazian H et al. Predictive role of radiomics features extracted from preoperative cross-sectional imaging of pancreatic ductal adenocarcinoma in detecting lymph node metastasis: a systemic review and meta-analysis. Abdom Radiol. 2023; 1–15. 10.1007/s00261-023-03940-y 37202642

[20] Salameh J-P, Bossuyt PM, McGrath TA et al. Preferred reporting items for systematic review and meta-analysis of diagnostic test accuracy studies (PRISMA-DTA): explanation, elaboration, and checklist. BMJ. 2020; 370. 10.1136/bmj.m2632 32816740

[21] Tabnak P, HajiEsmailPoor Z, Baradaran B et al. MRI-Based Radiomics methods for Predicting Ki-67 expression in breast Cancer: a systematic review and Meta-analysis. Acad Radiol. 2023. 10.1016/j.acra.2023.10.010 37925343

[22] Lambin P, Leijenaar RTH, Deist TM, et al. Radiomics: the bridge between medical imaging and personalized medicine. Nat Rev Clin Oncol. 2017;14:749–62. 28975929 10.1038/nrclinonc.2017.141

[23] Li X, Liu Q, Hu B, et al. A computed tomography-based clinical-radiomics model for prediction of lymph node metastasis in esophageal carcinoma. J Cancer Res Ther. 2021;17:1665–71. 35381737 10.4103/jcrt.jcrt_1755_21

[24] Shen C, Liu Z, Wang Z, et al. Building CT Radiomics Based Nomogram for Preoperative Esophageal Cancer patients Lymph Node Metastasis Prediction. Transl Oncol. 2018;11:815–24. 29727831 10.1016/j.tranon.2018.04.005 PMC6154864

[25] Ou J, Wu L, Li R, et al. CT radiomics features to predict lymph node metastasis in advanced esophageal squamous cell carcinoma and to discriminate between regional and non-regional lymph node metastasis: a case control study. Quant Imaging Med Surg. 2021;11:628–40. 33532263 10.21037/qims-20-241 PMC7779921

[26] Ding M, Cui H, Li B, et al. Integrating preoperative CT and clinical factors for Lymph Node Metastasis Prediction in Esophageal Cancer by Feature-wise Attentional Graph Neural Network (FAGNN). Int J Radiat Oncol Biol Phys. 2021;111:e123–4. 10.1016/j.ijrobp.2021.07.545 36641040

[27] Zhang C, Shi Z, Kalendralis P et al. Prediction of lymph node metastases using pretreatment PET radiomics of the primary tumour in esophageal adenocarcinoma: an external validation study. Br J Radiol. 2021; 94. 10.1259/bjr.20201042 PMC7934292 33264032

[28] Chen L, Ouyang Y, Liu S et al. Radiomics Analysis of Lymph Nodes with Esophageal Squamous Cell Carcinoma Based on Deep Learning. J Oncol. 2022; 2022. 10.1155/2022/8534262 PMC9489385 36147442

[29] Peng G, Zhan Y, Wu Y et al. Radiomics models based on CT at different phases predicting lymph node metastasis of esophageal squamous cell carcinoma (GASTO-1089). Front Oncol. 2022; 12. 10.3389/fonc.2022.988859 PMC9643555 36387160

[30] Tan X, Ma Z, Yan L, et al. Radiomics Nomogram outperforms size criteria in discriminating lymph node metastasis in resectable esophageal squamous cell carcinoma. Eur Radiol. 2019;29:392–400. 29922924 10.1007/s00330-018-5581-1

[31] Qu J, Shen C, Qin J, et al. The MR radiomic signature can predict preoperative lymph node metastasis in patients with esophageal cancer. Eur Radiol. 2019;29:906–14. 30039220 10.1007/s00330-018-5583-z

[32] Matsuda S, Takeuchi M, Kawakubo H, Kitagawa Y. Lymph node metastatic patterns and the development of multidisciplinary treatment for esophageal cancer. Dis Esophagus. 2023;36:doad006. 36857594 10.1093/dote/doad006 PMC10061432

[33] Shi Y, Xu J, Wang Y, et al. Prognostic significance of preoperative lymph node assessment for patients with stage pn0 esophageal squamous cell carcinoma after esophagectomy. J Thorac Dis. 2019;11:732–43. 31019761 10.21037/jtd.2019.02.25 PMC6462721

[34] Ibrahim A, Primakov S, Beuque M, et al. Radiomics for precision medicine: current challenges, future prospects, and the proposal of a new framework. Methods. 2021;188:20–9. 32504782 10.1016/j.ymeth.2020.05.022

[35] Bedrikovetski S, Dudi-Venkata NN, Kroon HM, et al. Artificial intelligence for pre-operative lymph node staging in colorectal cancer: a systematic review and meta-analysis. BMC Cancer. 2021;21:1–10. 34565338 10.1186/s12885-021-08773-w PMC8474828

[36] Wan Q, Zhou J, Xia X, et al. Diagnostic performance of 2D and 3D T2WI-based radiomics features with machine learning algorithms to distinguish solid solitary pulmonary lesion. Front Oncol. 2021;11:683587. 34868905 10.3389/fonc.2021.683587 PMC8637439

[37] Jia Q, Xu J, Jiang W, et al. Dynamic contrast-enhanced MR imaging in a phase II study on neoadjuvant chemotherapy combining Rh-endostatin with docetaxel and epirubicin for locally advanced breast cancer. Int J Med Sci. 2012;10:110–8. 23329881 10.7150/ijms.5123 PMC3547207

[38] Shambhu S, Koundal D, Das P, Sharma C. Binary classification of covid-19 ct images using cnn: Covid diagnosis using ct. Int J E-Health Med Commun. 2021;13:1–13. 10.4018/IJEHMC.20220701.oa4

[39] Shambhu S, Koundal D, Das P et al. Computational methods for automated analysis of malaria parasite using blood smear images: recent advances. Comput Intell Neurosci. 2022; 2022. 10.1155/2022/3626726 PMC9017520 35449742

[40] Shambhu S, Koundal D. Recent trends in image processing using granular computing. International Conference on Advanced Communication and Computational Technology, Springer. 2019, 469–479.

[41] Shambhu S, Koundal D, Das P. Deep learning-based computer assisted detection techniques for malaria parasite using blood smear images. Int J Adv Technol Eng Explor. 2023;10:990.

[42] Liu Y, Wei X, Zhang X, et al. CT radiomics combined with clinical variables for predicting the overall survival of hepatocellular carcinoma patients after hepatectomy. Transl Oncol. 2022;26:101536. 36115077 10.1016/j.tranon.2022.101536 PMC9483805

[43] Xu Y-H, Lu P, Gao M-C, et al. Progress of magnetic resonance imaging radiomics in preoperative lymph node diagnosis of esophageal cancer. World J Radiol. 2023;15:216. 37545645 10.4329/wjr.v15.i7.216 PMC10401402

[44] Huo R, Liu Y, Xu H, et al. Associations between carotid atherosclerotic plaque characteristics determined by magnetic resonance imaging and improvement of cognition in patients undergoing carotid endarterectomy. Quant Imaging Med Surg. 2022;12:2891. 35502372 10.21037/qims-21-981 PMC9014142

[45] Zhao X, Zhang G, Chen J et al. A rationally designed nuclei-targeting FAPI 04-based molecular probe with enhanced tumor uptake for PET/CT and fluorescence imaging. Eur J Nucl Med Mol Imaging. 2024; 1–12. 10.1007/s00259-024-06691-0 38512485

[46] Zharmagambetov A, Gabidolla M, Carreira-Perpinán MA. Improved multiclass AdaBoost for image classification: The role of tree optimization. 2021 IEEE International Conference on Image Processing (ICIP), IEEE. 2021, 424–428.

[47] Zheng J, Lin D, Gao Z, et al. Deep learning assisted efficient AdaBoost algorithm for breast cancer detection and early diagnosis. IEEE Access. 2020;8:96946–54. 10.1109/ACCESS.2020.2993536

[48] Giglio C, Brown SD. Using elastic net regression to perform spectrally relevant variable selection. J Chemom. 2018;32:e3034. 10.1002/cem.3034

[49] Vinga S. Structured sparsity regularization for analyzing high-dimensional omics data. Brief Bioinform. 2021;22:77–87. 32597465 10.1093/bib/bbaa122

[50] Mao K, Wong LM, Zhang R, et al. Radiomics Analysis in characterization of salivary gland tumors on MRI: a systematic review. Cancers (Basel). 2023;15:4918. 37894285 10.3390/cancers15204918 PMC10605883

[51] Kocak B, Akinci D’Antonoli T, Mercaldo N, et al. METhodological RadiomICs score (METRICS): a quality scoring tool for radiomics research endorsed by EuSoMII. Insights Imaging. 2024;15:8. 38228979 10.1186/s13244-023-01572-w PMC10792137

[52] Kocak B, Baessler B, Bakas S, et al. CheckList for EvaluAtion of Radiomics research (CLEAR): a step-by-step reporting guideline for authors and reviewers endorsed by ESR and EuSoMII. Insights Imaging. 2023;14:75. 37142815 10.1186/s13244-023-01415-8 PMC10160267

[53] Doniselli FM, Pascuzzo R, Mazzi F et al. Quality assessment of the MRI-radiomics studies for MGMT promoter methylation prediction in glioma: a systematic review and meta-analysis. Eur Radiol. 2024; 1–14. 10.1007/s00330-024-10594-x PMC11364578 38308012

[54] Abbaspour E, Karimzadhagh S, Monsef A, et al. Application of radiomics for preoperative prediction of lymph node metastasis in colorectal cancer: a systematic review and Meta-analysis. Int J Surg. 2024;10:1097. 10.1097/JS9.0000000000001239 PMC11175807 38935817

