# Correction: Radiomics diagnostic performance for predicting lymph node metastasis in esophageal cancer: a systematic review and meta-analysis

**DOI:** 10.1186/s12880-024-01411-4

**Published:** 2024-08-28

**Authors:** Dong Ma, Teli Zhou, Jing Chen, Jun Chen

**Affiliations:** 1 The Fifth Affiliated Hospital, Southern Medical University, Guangzhou, 510900 Guangdong China; 2 Guangzhou Shiyuan Clinics Co., Ltd, Guangzhou, 510530 Guangdong China; 3 Dingxi People’s Hospital, Dingxi, Gansu, 743000 China

**Correction to: BMC Medical Imaging (2024) 24:144**

10.1186/s12880-024-01278-5

Following the publication of the Original Article, the authors reported an error in the affiliations of two authors.

**Incorrect**:

Teli Zhou

The Fifth Affiliated Hospital, Southern Medical University, Guangzhou 510,900, Guangdong, China; and Yibicom Health Management, Guangzhou, Guangdong 510,700, China.

Jun Chen

The Fifth Affiliated Hospital, Southern Medical University, Guangzhou 510,900, Guangdong, China.

**Correct**:

Teli Zhou

Guangzhou Shiyuan Clinics Co., Ltd, Guangzhou 510,530, Guangdong, China.

Jun Chen

Dingxi People’s Hospital, Dingxi 743,000, Gansu, China.

The affiliations have been updated above.

The Original Article has been corrected.

