# Supplementary material for: Radiomics diagnostic performance for predicting lymph node metastasis in esophageal cancer: a systematic review and meta-analysis
Source: BMC Med Imaging. 2024 Jun 12;24:144. doi: 10.1186/s12880-024-01278-5 (PMC11170881; doi:10.1186/s12880-024-01278-5)
Supplement: Supplementary file 1 — Supplementary Material 1 [file 12880_2024_1278_MOESM1_ESM.docx]

Table 1- Embase 16/11/2023

| Set | Query | Records |
| --- | --- | --- |
| #1 | 'lymph node metastasis'/exp OR 'lymph node metastasis' OR 'lymph node metastases'/exp OR 'lymph node metastases' OR 'lnm' | 192,235 |
| #2 | 'radiomics'/exp OR 'radiomics' OR 'machine learning'/exp OR 'machine learning' OR 'artificial intelligence'/exp OR 'artificial intelligence' OR 'deep learning'/exp OR 'deep learning' OR 'nomogram'/exp OR 'nomogram' OR 'radiomics model'/exp OR 'radiomics model' OR 'texture analysis'/exp OR 'texture analysis' OR 'feature extraction'/exp OR 'feature extraction' | 539,707 |
| #4 | 'esophagus tumor'/exp OR 'esophagus tumor' OR 'esophagus cancer'/exp OR 'esophagus cancer' OR 'esophageal cancer'/exp OR 'esophageal cancer' OR 'esophageal tumor'/exp OR 'esophageal tumor' OR 'esophageal adenocarcinoma'/exp OR 'esophageal adenocarcinoma' OR 'esophageal squamous cell carcinoma'/exp OR 'esophageal squamous cell carcinoma' OR 'oesophagus tumor'/exp OR 'oesophagus tumor' OR 'oesophagus cancer'/exp OR 'oesophagus cancer' OR 'oesophageal cancer'/exp OR 'oesophageal cancer' OR 'oesophageal tumor'/exp OR 'oesophageal tumor' OR 'oesophageal adenocarcinoma'/exp OR 'oesophageal adenocarcinoma' OR 'oesophageal squamous cell carcinoma'/exp OR 'oesophageal squamous cell carcinoma' | 117,548 |
| Combined set | **#1 AND #2 AND #3** | 218 |

Table 2-PubMed

| Set | Query | Records |
| --- | --- | --- |
| #1 | (lymph node metastasis) OR (lymph node metastases) OR (LNM) OR ((lymph) AND (node) AND (metastasis)) | 151,899 |
| #2 | **(Radiomics) OR (machine learning) OR (machine AND learning) OR (deep learning) OR (deep AND learning) OR (neural network) OR (neural AND network) OR (radiomics nomogram) OR (Artificial Intelligence) OR (Artificial AND Intelligence) OR (texture analysis) OR (feature AND extract*) OR (texture AND analysis)** | 455,421 |
| #3 | (((((((((((((esophagus tumor) OR (esophagus cancer)) OR (esophageal cancer)) OR (esophageal tumor)) OR (esophageal adenocarcinoma)) OR (esophageal adenocarcinoma)) OR (esophageal squamous cell carcinoma)) OR (oesophagus tumor)) OR (oesophagus cancer)) OR (oesophageal cancer)) OR (oesophageal tumor)) OR (oesophageal adenocarcinoma)) OR (oesophageal adenocarcinoma)) OR (oesophageal squamous cell carcinoma) | 91,112 |
| Combined set | **#1 AND #2 AND #3** | 67 |

Table 3- Web of Science

| Set | Query | Records |
| --- | --- | --- |
| #1 | (((((((((((((ALL=(esophagus tumor )) OR ALL=(esophagus cancer)) OR ALL=(esophageal cancer)) OR ALL=(esophageal tumor)) OR ALL=(esophageal adenocarcinoma)) OR ALL=(esophageal adenocarcinoma)) OR ALL=(esophageal squamous cell carcinoma)) OR ALL=(oesophagus tumor)) OR ALL=(oesophagus cancer)) OR ALL=(oesophageal cancer)) OR ALL=(oesophageal tumor)) OR ALL=(oesophageal adenocarcinoma)) OR ALL=(oesophageal adenocarcinoma)) OR ALL=(oesophageal squamous cell carcinoma) | 81,954 |
| #2 | (((((((ALL=(radiomics)) OR ALL=(machine learning)) OR ALL=(artificial intelligence)) OR ALL=(deep learning)) OR ALL=(nomogram)) OR ALL=(radiomics model)) OR ALL=(texture analysis)) OR ALL=(feature extraction) | 728,731 |
| #3 | ((ALL=(lymph node metastasis)) OR ALL=(lymph node metastases)) OR ALL=(LNM) | 97,128 |
| Combined set | **#1 AND #2 AND #3 AND** | 141 |

Table S4- Modified signaling questions of QUADAS-2 tool.

| 1. Risk of Bias | | | |
| --- | --- | --- | --- |
| Patient Selection | Index Test | Reference standard | Flow and Timing |
| - Were the inclusion/exclusion criteria specified? - Was the type of study specified (Retro vs Pro)? - Were patients treatment-naive? | - Where imaging acquisition protocol and segmentation method detailed? - Was the image processing approach detailed? - Was a validation method used? | - Is the reference standards likely to correctly classify the target condition? | - Was there an appropriate interval between index test and reference standard? - Did all patients receive the same reference standard? |
| 1. Concerns Regarding Applicability | | | |
| Patient Selection | Index Test | Reference standard | |
| - Are there concerns that the included patients and setting do not match the review question? | - Are there concerns that the index test, its conduct, or its interpretation differ from the review question? | - Are there concerns that the target condition, as defined by the reference standard, does not match the question? | |
